# Compositional synthesis of modular systems

**DOI:** 10.1007/s11334-022-00450-w

**Published:** 2022-04-01

**Authors:** Bernd Finkbeiner, Noemi Passing

**Affiliations:** CISPA Helmholtz Center for Information Security, Saarbrücken, Germany

**Keywords:** Compositional synthesis, Bounded synthesis, Reactive systems, Distributed systems

## Abstract

In contrast to the breakthroughs in reactive synthesis of monolithic systems, distributed synthesis is not yet practical. Compositional approaches can be a key technique for scalable algorithms. Here, the challenge is to decompose a specification of the global system into local requirements on the individual processes. In this paper, we present and extend a sound and complete compositional synthesis algorithm that constructs for each process, in addition to the strategy, a certificate that captures the necessary interface between the processes. The certificates define an assume-guarantee contract that allows for formulating individual process requirements. By bounding the size of the certificates, we then bias the synthesis procedure towards solutions that are desirable in the sense that they have a small interface. We have implemented our approach and evaluated it on scalable benchmarks: It is much faster than standard methods for distributed synthesis as long as reasonably small certificates exist. Otherwise, the overhead of synthesizing additional certificates is small.

## Introduction

In spite of the recent advances in practical reactive synthesis for monolithic systems, no scalable tools for the synthesis from arbitrary distributed system architectures exist. In verification, compositionality, i.e., breaking down the verification of a complex system into several smaller tasks over individual components, proved to be a key technique for scalable algorithms [8].

Developing compositional approaches for synthesis, however, is much more challenging: In practice, an individual process can rarely guarantee the satisfaction of the specification alone. Typically, there are input sequences preventing a process from satisfying the specification and the other processes in the system ensure that these sequences are not produced. Thus, a process needs information about the behavior of the other processes and therefore distributed synthesis cannot easily be broken down into tasks over the individual processes.

In this paper, we present and extend *certifying synthesis* [14], a compositional synthesis algorithm that constructs additional *guarantees on the behavior* of every process. These so-called *certificates* then define an assume-guarantee contract: A strategy is only required to satisfy the specification if the other processes do not deviate from their guaranteed behavior. This allows for considering a process independent of the other processes’ strategies. Our algorithm is an extension of bounded synthesis [16] that incorporates the search for certificates into the synthesis task for the strategies.

Synthesizing additional certificates has several benefits: Since the strategies only depend on the other processes’ certificates and not on their particular strategies, certifying synthesis *enables modularity of the system*: The certificates form a contract between the processes. Once the contract has been synthesized, strategies can be exchanged safely with other ones respecting the contract. Thus, strategies can be adapted flexibly without synthesizing a solution for the whole system again if requirements that do not affect the contract change.

Moreover, the certificates capture which information a process needs about the behavior of the other processes to be able to satisfy the specification while abstracting from their irrelevant behavior. Certifying synthesis thus allows for analyzing strategies locally and for *recognizing the system’s interconnections*.

Certifying synthesis introduces bounds on the sizes of the certificates. Hence, it bounds the size of the interface between the processes. By starting with small bounds and by only increasing them if the specification is unrealizable for the given ones, our algorithm restricts synthesis to search for solutions with small interfaces which are often desired in practice. Thus, certifying synthesis *guides the synthesis procedure*.

We present two representations of certificates, as LTL formulas and as labeled transition systems. We prove soundness and completeness of certifying synthesis for both of them. Moreover, we extend the latter representation with nondeterminism, permitting smaller certificates for certain specifications. We present an optimization of certifying synthesis that reduces the number of considered certificates by determining *relevant processes* for each process. Soundness and completeness are preserved. Focusing on transition system certificates, both deterministic and nondeterministic, we present an algorithm for synthesizing certificates that is based on a reduction to a SAT constraint system.

We implemented the algorithm and compare it to an extension [2] of the bounded synthesis tool BoSy [10] to distributed systems and to a compositional synthesis algorithm based on dominant strategies [7]. The results clearly demonstrate the advantage of synthesizing certificates: If solutions with a small interface between the processes exist, our algorithm outperforms the other ones significantly. Otherwise, the overhead of synthesizing certificates is small. Permitting nondeterminism can reduce the strategy and certificate sizes notably.

*Related work* There are several compositional synthesis approaches for monolithic systems [11–13, 18, 19]. We, however, focus on distributed synthesis algorithms. Assume-guarantee synthesis [5] is closest to our approach. There, each process provides a guarantee on its own behavior and, in return, makes an assumption on the behavior of the other processes. If, for each process, there is a strategy that satisfies the specification under the hypothesis that the other processes respect the assumption, and if the guarantee implies the assumptions of the other processes, a solution for the whole system is found. In contrast to our approach, most assume-guarantee synthesis algorithms [1, 3–5] either rely on the user to provide the assumptions or require that a strategy profile on which the strategies can synchronize is constructed prior to synthesis.

Assume-guarantee distributed synthesis [21] synthesizes assume-guarantee contracts using a negotiation algorithm. In contrast to our approach, the synthesized guarantees do not necessarily imply the assumptions of the other processes. Thus, the assumptions and guarantees need to be iteratively refined until a valid contract is found and therefore the algorithm is not complete. This iteration is circumvented in our approach since the assumptions are constructed from the certificates.

Using a weaker winning condition for synthesis, remorse-free dominance [6], avoids the explicit construction of assumptions and guarantees [7]. Yet, the implicit assumptions do not always suffice. Thus, although a dependency analysis of the specification allows for solutions for further systems and specifications [13], compositional solutions do not always exist.

## Running example

In many modern factories, autonomous robots are a crucial component in the production line. Since the correctness of their implementation is essential, they are a natural target for synthesis. Consider a factory with two robots that carry production parts from one machine to another. There is a crossing that is used by both robots. The robots are required to prevent a crash at the crossing: , where $at\_c _{i}$ is an input variable denoting that robot $r_i$ arrived at the crossing, and $\mathrm{go}_{i}$ is an output variable of robot $r_i$ denoting that $r_i$ moves ahead. Moreover, both robots need to cross the intersection at some point in time after arriving there: . In addition, both robots have further objectives $\varphi _{\mathrm{add}_i}$ that are specific to their area of application. For instance, they may capture which machines have to be approached.

None of the robots can satisfy $\varphi _{\mathrm{safe}} \wedge \varphi _{\mathrm{cross}_i}$ alone: No matter when $r_i$ enters the crossing, $r_j$ might enter it at the same time. Thus, strategies cannot be synthesized individually without information on the other robot’s behavior. Due to $\varphi _{\mathrm{add}_i}$, the parallel composition of the strategies can be large and complex. Hence, understanding why the overall specification is met and recognizing the individual strategies is challenging.

If the robots commit to their behavior at crossings, however, individual solutions can be found. For instance, if $r_2$ guarantees to always give priority to $r_1$, a strategy for $r_1$ that enters crossings regardless of $r_2$ satisfies $\varphi _{\mathrm{safe}}\wedge \varphi _{\mathrm{cross}_1}$ since $r_1$ may assume that $r_2$ will not deviate from its certificate. If $r_1$ guarantees to not block crossings, $r_2$ can satisfy $\varphi _{\mathrm{safe}}\wedge \varphi _{\mathrm{cross}_2}$ as well. Since the assumptions of the robots are constructed from the certificates and thus from the guaranteed behavior, the parallel composition of the robots’ strategies satisfies the whole specification as long as the strategies satisfy the additional requirements $\varphi _{{\mathrm{add}_i}}$ as well.

Furthermore, we then know that the robots solely interfere at crossings. Thus, the certificates provide insight in the required communication of the robots and abstract away the irrelevant behavior, i.e., the behavior aside from crossings, of the other robot. Particularly for large $\varphi _{\mathrm{add}_i}$, this significantly increases the understandability of why $r_i$’s strategy satisfies $\varphi _i$. Moreover, the certificates form a contract of safe behavior at crossings: If $\varphi _{\mathrm{add}_i}$ changes, it suffices to synthesize a new strategy for $r_i$. Provided $r_i$ does not change its behavior at crossings, $r_j$’s strategy can be left unchanged.

Throughout this paper, we explain the introduced concepts and formalisms using the above example. The individual explanations, that are all marked with “running example”, can then be used to retrace the whole synthesis procedure for the manufacturing robots. Furthermore, we provide a summary of the individual steps for certifying synthesis with deterministic certificates together with the synthesized strategies and certificates at the end of Sect. 5.

## Preliminaries

*Notation.* We denote the prefix of length *t* of an infinite word $\sigma = \sigma _1 \sigma _2 \ldots \in (2^V)^\omega$ by $\sigma _{| t} := \sigma _{1} \ldots \sigma _{t}$. For a set *X* and an infinite word $\sigma = \sigma _1 \sigma _2 \ldots \in (2^V)^\omega$, we define $\sigma \cap X = (\sigma _1 \cap X)(\sigma _2 \cap X)\ldots \in (2^X)^\omega$.

*LTL.* Linear-time temporal logic (LTL) [22] is a specification language for linear-time properties. For a finite set of atomic propositions $\Sigma$ and $a \in \Sigma$, the syntax of LTL is given byWe define the temporal operators  and  and use the standard semantics. The language $\mathcal {L}(\varphi )$ of a formula $\varphi$ is the set of infinite words that satisfy $\varphi$. The atomic propositions in $\varphi$ are denoted by ${\text {prop}}(\varphi )$. We represent a formula $\varphi = \xi _1 \wedge \ldots \wedge \xi _k$ also by the set of its conjuncts, i.e., $\varphi = \{\xi _1, \ldots , \xi _k\}$.

*Automata.* Given a finite alphabet $\Sigma$, a universal co-Büchi automaton $\mathcal {A} = (Q,q_0,\delta ,F)$ over $\Sigma$ consists of a finite set of states *Q*, an initial state $q_0 \in Q$, a transition relation $\delta : Q \times 2^\Sigma \times Q$, and a set $F \subseteq Q$ of rejecting states. For an infinite word $\sigma = \sigma _0\sigma _1 \ldots \in (2^\Sigma )^\omega$, a run of $\sigma$ on $\mathcal {A}$ is an infinite sequence $q_0 q_1 \ldots \in Q^\omega$ of states with $(q_i,\sigma _i,q_{i+1}) \in \delta$ for all $i \ge 0$. A run is called accepting if it contains only finitely many visits to rejecting states. $\mathcal {A}$ accepts a word $\sigma$ if all runs of $\sigma$ on $\mathcal {A}$ are accepting. The language $\mathcal {L}(\mathcal {A})$ of $\mathcal {A}$ is the set of all accepted words. An LTL formula $\varphi$ can be translated into an equivalent universal co-Büchi automaton $\mathcal {A}_\varphi$, i.e., and automaton with $\mathcal {L}(\varphi ) = \mathcal {L}(\mathcal {A}_\varphi )$, with a single exponential blow up [20].

*Architectures.* An architecture *A* is a tuple $(P, V, {I}, {O})$, where *P* is a set of processes consisting of the environment process $\mathrm{env}$ and a set of *n* system processes $P^-\!= P {\setminus }\{\mathrm{env}\}$, *V* is a set of variables, ${I}= \langle I_1, \ldots , I_n \rangle$ assigns a set ${I_{j}} \subseteq V$ of input variables to each system process $p_j$, and ${O}= \langle O_\mathrm{env}, O_1, \ldots O_n \rangle$ assigns a set ${O_{j}} \subseteq V$ of output variables to each process $p_j$. For all $p_j, p_k \in P^-\!$ with $j \ne k$, we have ${I_{j}} \cap {O_{j}} = \emptyset$ and ${O_{j}} \cap {O_{k}} = \emptyset$. The variables ${V_{j}}$ of $p_j \in P^-\!$ are its inputs and outputs, i.e., ${V_{j}} = {I_{j}} \cup {O_{j}}$. The variables *V* of the whole system are defined by $V = \bigcup _{p_j \in P^-\!} {V_{j}}$. We define $\mathrm{inp}= \bigcup _{p_j \in P^-\!} {I_{j}}$ and $\mathrm{out}= \bigcup _{p_j \in P^-\!} {O_{j}}$. An architecture is called distributed if $|P^-\!| \ge 2$ and monolithic otherwise. In the remainder of this paper, we assume that a distributed architecture is given.

*Labeled Transition Systems.* For sets *I* and *O* of input and output variables, a Moore transition system (TS) $\mathcal {T}~= (T,t_0,\tau ,o)$ consists of a finite set of states *T*, an initial state $t_0$, a transition function $\tau : T \times 2^I \rightarrow T$, and a labeling function $o: T \rightarrow 2^O$. For an input sequence $\gamma = \gamma _0 \gamma _1 \ldots c \in (2^{I})^\omega$, $\mathcal {T}$ produces a path

$$\begin{aligned}\pi = (t_0 , \gamma _0 \cup o(t_0)) (t_1 , \gamma _1 \cup o(t_1)) \ldots c \in (T \times 2^{I \cup O})^\omega ,\end{aligned}$$

where $(t_j, \gamma _j,t_{j+1}) \in \tau$. The projection of a path to the variables is called trace. The parallel composition of two TS $\mathcal {T}_1 = (T_1,t^1_0,\tau _1,o_1)$ and $\mathcal {T}_2 = (T_2,t^2_0,\tau _2,o_2)$, is given by $\mathcal {T}_1 {{\,\mathrm{||}\,}}\mathcal {T}_2 = (T,t_0,\tau ,o)$, with

- $$T = T_1 \times T_2$$,
- $$t_0=(t^1_0,t^2_0)$$
- $$\tau ((t,t'),\varvec{i}) = (\tau _1(t,\varvec{i} \cap {I_{1}}),\tau _2(t',\varvec{i}\cap {I_{2}}))$$, and
- $$o((t,t')) = o_1(t) \cup o_2(t')$$.

A TS $\mathcal {T}_1 = (T_1,t^1_0,\tau _1,o_1)$ over *I* and $O_1$ *simulates* a TS $\mathcal {T}_2 = (T_2,t^2_0,\tau _2,o_2)$ over *I* and $O_2$ with $O_1 \subseteq O_2$, denoted $\mathcal {T}_2 \preceq \mathcal {T}_1$, if there exists a simulation relation $R: T_2 \times T_1$ with

- $$(t^2_0,t^1_0)\in R$$,
- $o_2(t_2) \cap O_1 = o_1(t_1)$ for all $(t_2,t_1)\in R$, and
- For all $t'_2 \in T_2$, $\varvec{i} \in 2^I$, if $\tau _2(t_2,\varvec{i}) = t'_2$, then there is some $t'_1 \in T_1$ such that $\tau _1(t_1,\varvec{i})=t'_1 \wedge (t'_2,t'_1) \in R$.

*Strategies.* We model a strategy $s_i$ of process $p_i\in P^-\!$ as a Moore transition system $\mathcal {T}_i$ over ${I_{i}}$ and ${O_{i}}$. The trace produced by $\mathcal {T}_i$ on $\gamma \in (2^{{I_{i}}})^\omega$ is called the *computation* of $s_i$ on $\gamma$, denoted $\mathrm{comp}(s_i,\gamma )$. For an LTL formula $\varphi$ over *V*, $s_i$ satisfies $\varphi$, denoted $s_i \models \varphi$, if we have $\mathrm{comp}(s,\gamma ) \cup \gamma ' \models \varphi$ for all $\gamma \in (2^{{I_{i}}})^\omega$, $\gamma ' \in (2^{V{\setminus }{V_{i}}})^\omega$.

*Synthesis.* Given a formal specification $\varphi$, synthesis derives strategies $s_1, \ldots , s_n$ for the system processes such that $s_1 {{\,\mathrm{||}\,}}\cdots {{\,\mathrm{||}\,}}s_n \models \varphi$ holds. If such strategies exist, $\varphi$ is called realizable. Bounded synthesis [16] additionally bounds the size of the strategies. The search for strategies of a certain size is encoded into a constraint system that is satisfiable if, and only if, $\varphi$ is realizable for the bound. There are SMT, SAT, QBF, and DQBF encodings for monolithic [9] and distributed [2] architectures.

## Compositional synthesis with certificates

In this section, we present a sound and complete compositional synthesis algorithm for distributed systems. The main idea is to synthesize strategies for the system processes individually. Thus, in contrast to classical distributed synthesis, where strategies $s_1, \ldots , s_n$ are synthesized such that $s_1 {{\,\mathrm{||}\,}}\cdots {{\,\mathrm{||}\,}}s_n \models \varphi$ holds, we require that $s_i \models \varphi _i$ holds for all $p_i \in P^-\!$. Here, $\varphi _i$ is a subformula of $\varphi$ that captures the parts of $\varphi$ affecting $p_i$. As long as $\varphi _i$ contains all parts of $\varphi$ that restrict the behavior of $s_i$, the satisfaction of $\varphi$ by $s_1 {{\,\mathrm{||}\,}}\cdots {{\,\mathrm{||}\,}}s_n$ is guaranteed. Computing specification decompositions is not the main focus of this paper; in fact, our algorithm can be used with any decomposition fulfilling the above requirement. There is work on obtaining small subspecifications, e.g., [12], we, however, use an easy decomposition in the following for simplicity:

### Definition 4.1

*(Specification decomposition)* Let $\varphi = \xi _1 \wedge \ldots \wedge \xi _k$ be an LTL formula. The *decomposition of* $\varphi$ is a vector $\langle \varphi _1, \ldots , \varphi _n \rangle$ of LTL formulas with $\varphi _i = \{ \xi _j \in \varphi \mid {\text {prop}}(\xi _j) \cap {O_{i}} \ne \emptyset \,\vee \, {\text {prop}}(\xi _j) \cap \mathrm{out}= \emptyset \}$.

Intuitively, the subspecification $\varphi _i$ contains all conjuncts of $\varphi$ that contain outputs of $p_i$ as well as all input-only conjuncts. In the remainder of this paper, we assume that both ${\text {prop}}(\varphi ) \subseteq V$ and $\mathcal {L}(\varphi ) \in (2^V)^\omega$ hold for all specifications $\varphi$. Then, every atomic proposition occurring in $\varphi$ is an input or output of at least one system process and thus $\bigwedge _{p_i\in P^-\!} \varphi _i = \varphi$ holds.

### Running example

Recall the robots from Sect. 2 and assume for simplicity that both do not have any additional objectives $\varphi _{\mathrm{add}_i}$. Thus, the overall specification is given by $\varphi = \varphi _{\mathrm{safe}} \wedge \varphi _{\mathrm{cross}_1} \wedge \varphi _{\mathrm{cross}_1}$. Then, we obtain the subspecifications $\varphi _i = \varphi _{\mathrm{safe}} \wedge \varphi _{\mathrm{cross}_i}$ with Definition 4.1 since $\varphi _{\mathrm{cross}_{1-i}}$ does not contain any output variables of $r_i$, while $\varphi _{\mathrm{safe}}$ and $\varphi _{\mathrm{cross}_i}$ clearly do.

Although we decompose the specification, a process $p_i$ usually cannot guarantee the satisfaction of $\varphi _i$ alone; rather, it depends on the cooperation of the other processes. For instance, robot $r_1$ from Sect. 2 cannot guarantee that no crash will occur when entering the crossing since $r_2$ can enter it at the same point in time. Thus, we additionally synthesize a *guarantee on the behavior* of each process, the so-called *certificate*. The certificates then provide essential information to the processes: If $p_i$ commits to a certificate, the other processes can rely on $p_i$’s strategy to not deviate from this behavior. In particular, the strategies only need to satisfy the specification as long as the other processes stick to their certificates. Thus, a process is not required to react to *all* behaviors of the other processes but only to those that truly occur when the processes interact. In this section, we represent the certificate of a process $p_i$ by an LTL formula $\psi _{i}$.

### Running example

If robot $r_2$ guarantees to always give priority to $r_1$ at crossings, its LTL certificate can be given by , for instance. Since $r_1$ can assume that $r_2$ does not deviate from its certificate $\psi _2$, a strategy for $r_1$ that enters crossings regardless of $r_2$ satisfies $\varphi _{\mathrm{safe}} \wedge \varphi _{\mathrm{cross}_1}$.

To ensure that $p_i$ does not deviate from its own certificate $\psi _{i}$, we require its strategy $s_i$ to satisfy $\psi _{i}$. To model that $s_i$ only has to satisfy its specification if the other processes stick to their certificates, it has to satisfy $\Psi _{i} \rightarrow \varphi _i$, where $\Psi _{i} = \{ \psi _{j} \mid p_j \in P^-\!{\setminus }\{p_i\}\}$, i.e., $\Psi _{i}$ is the conjunction of the certificates of the other processes. Using this, we define certifying synthesis:

### Definition 4.2

*(Certifying synthesis)* Let $\varphi$ be an LTL formula. *Certifying synthesis* for $\varphi$ derives vectors $\mathcal {S} = \langle s_1,\ldots ,s_n \rangle$ and $\Psi = \langle \psi _{1},\ldots ,\psi _{n} \rangle$ of strategies and LTL certificates, respectively, for the system processes such that $s_i \models \psi _{i} \wedge (\Psi _{i} \rightarrow \varphi _i)$ for all $p_i \in P^-\!$, where $\Psi _{i} = \{ \psi _{j} \mid p_j \in P^-\!{\setminus }\{p_i\}\}$. Then, $(\mathcal {S},\Psi )$ *realizes* $\varphi$.

Classical algorithms for distributed synthesis reason *globally* about the satisfaction of the specification by the parallel composition of the synthesized strategies. Certifying synthesis, in contrast, reasons *locally* about the satisfaction of the subspecifications for the individual processes, i.e., without considering the composition of the strategies. Hence the strategies can be considered separately, greatly improving the understandability of the synthesized solutions. Moreover, local reasoning is sound and complete. Intuitively, soundness follows from the fact that every system process is required to satisfy its own certificate. Completeness is obtained since every strategy can serve as its own certificate. Formally:

### Theorem 4.1

Let $\varphi$ be an LTL formula. Moreover, let $\mathcal {S} = \langle s_1, \ldots , s_n \rangle$ be a vector of strategies. There is a vector $\Psi = \langle \psi _{1}, \ldots , \psi _{n} \rangle$ of LTL certificates such that $(\mathcal {S},\Psi )$ realizes $\varphi$ if, and only if $s_1 {{\,\mathrm{||}\,}}\cdots {{\,\mathrm{||}\,}}s_n \models \varphi$ holds.

### Proof

Suppose that $(\mathcal {S},\Psi )$ realizes $\varphi$ for some $\Psi$. Let $\Psi _{i} = \{ \psi _{j} \mid p_j \in P^-\!{\setminus } \{p_i\} \}$. Let $\gamma \in (2^{{O_{\mathrm{env}}}})^\omega$ and $\sigma = \mathrm{comp}(s_1 {{\,\mathrm{||}\,}}\ldots {{\,\mathrm{||}\,}}s_n,\gamma )$. Since processes do not share outputs, $\mathrm{comp}(s_i,\sigma \cap {I_{i}})\cup (\sigma \cap (V{\setminus }{V_{i}})) = \sigma$ holds for all $p_i \in P^-\!$. By assumption, $s_i \models \psi _{i} \wedge (\Psi _{i} \rightarrow \varphi _i)$ holds for all $p_i \in P^-\!$. Hence, $\sigma \models \bigwedge _{1 \le i \le n} \psi _{i}$ and thus $\sigma \models \Psi _{i}$ for all $1 \le i \le n$. Therefore, $\sigma \models \bigwedge _{1 \le i \le n} \varphi _i$ follows. Since $\bigwedge _{1 \le i \le n} \varphi _i = \varphi$, we obtain $s_1 {{\,\mathrm{||}\,}}\cdots {{\,\mathrm{||}\,}}s_n \models \varphi$.

Let $s_1 {{\,\mathrm{||}\,}}\cdots {{\,\mathrm{||}\,}}s_n \models \varphi$. We construct LTL formulas $\psi _{i}$ that describe exactly the behavior of $s_i$, i.e., such that $\mathcal {L}(\psi _{i}) = \{ \mathrm{comp}(s_i,\gamma ) \cup \gamma ' \mid \gamma \in (2^{I_{i}})^\omega , \gamma ' \in (2^{V {\setminus } {V_{i}}})^\omega \}$. Since strategies are modeled with finite-state TS, such LTL formulas exist. Let $\Psi _{i} = \{ \psi _{j} \mid p_j \in P^-\!{\setminus } \{p_i\} \}$. It remains to show that $s_i \models \psi _{i} \wedge (\Psi _{i} \rightarrow \varphi _i)$ holds for all $1 \le i \le n$. Let $p_i \in P^-\!$. By construction of $\psi _{i}$, clearly $s_i \models \psi _{i}$ holds. Let $\gamma \in (2^{{I_{i}}})^\omega$, $\gamma ' \in (2^{V{\setminus }{V_{i}}})^\omega$ and let $\sigma = \mathrm{comp}(s_i,\gamma ) \cup \gamma '$. If $\sigma \models \lnot \Psi _{i}$, then $\sigma \models \Psi _{i} \rightarrow \varphi _i$ follows directly. Otherwise, i.e., if $\sigma \models \Psi _{i}$ holds, we have $\sigma \models \bigwedge _{1 \le i \le n} \psi _{i}$ by construction of $\Psi _{i}$ and since $s_i \models \psi _{i}$. Thus, $\sigma = \mathrm{comp}(s_1 {{\,\mathrm{||}\,}}\cdots {{\,\mathrm{||}\,}}s_n,\sigma \cap {O_{\mathrm{env}}})$ follows by construction of $\psi _{i}$. By definition, ${O_{i}} \cap {O_{\mathrm{env}}}= \emptyset$ holds and thus $\sigma \cap {O_{\mathrm{env}}}= (\gamma \cup \gamma ') \cap {O_{\mathrm{env}}}$. Hence, $\sigma = \mathrm{comp}(s_1 {{\,\mathrm{||}\,}}\cdots {{\,\mathrm{||}\,}}s_n, (\gamma \cup \gamma ')$. By assumption and since $\bigwedge _{1 \le i \le n} \varphi _i = \varphi$, we thus have $\sigma \models \varphi _i$ and, in particular, $\sigma \models \Psi _{i} \rightarrow \varphi _i$. Therefore, $s_i \models \Psi _{i} \rightarrow \varphi _i$ and hence, together with the previous result that $s_i \models \psi _{i}$ holds, we obtain $s_i \models \psi _{i} \wedge (\Psi _{i} \rightarrow \varphi _i)$ for all $p_i \in P^-\!$. $\square$

Certifying synthesis thus enables modularity and increases the understandability of the system due to local reasoning, while ensuring to find solutions for all specifications that are realizable in the architecture. Moreover, the parallel composition of the synthesized strategies is a correct solution for the whole system.

## Synthesis with deterministic certificates

There are several quality measures for certificates, for instance their size. We focus on certificates that are *easy to synthesize* in the sense that certifying synthesis can be integrated into existing synthesis algorithms. Therefore, we model certificates with labeled transition systems in the following. In this section, we restrict certificates to be deterministic. This avoids an exponential blowup due to determinization [23] when determining whether a strategy sticks to its own certificate. In Sect. 8, we lift this restriction and consider certificates modeled by nondeterministic transition systems. Note that while enforcing determinism may yield larger certificates, it does not rule out any strategy that can be found with nondeterministic certificates: Since strategies are per se deterministic, there exists at least one deterministic certificate for them: The strategy itself.

We model the certificate $g_i$ of a system process $p_i$ as a TS $\mathcal {T}^G_{i}$, called *guarantee transition system* (GTS), over inputs ${I_{i}}$ and *guarantee output variables* ${O^G_{i}}\subseteq {O_{i}}$. The *computation* of a GTS is the trace produced by it. Only considering a subset of ${O_{i}}$ as output variables allows the certificate to abstract from outputs of $p_i$ whose valuation is irrelevant for all other processes. In the following, we assume the guarantee output variables of $p_i$ to be both an output of $p_i$ and an input of some other process, i.e., ${O^G_{i}}\,{:}= {O_{i}} \cap \mathrm{inp}$. Intuitively, a variable $v \in {O_{i}} {\setminus } {O^G_{i}}$ cannot be observed by any other process. Thus, a guarantee on its behavior does not influence any process and hence it can be omitted. The variables ${V^G_{i}}$ of the GTS of $p_i$ are given by ${V^G_{i}}\,{:}= {I_{i}} \cup {O^G_{i}}$.

In certifying synthesis, it is crucial that a strategy only needs to satisfy the specification if the other processes do not deviate from their certificates. For LTL certificates, we use an implication in the local objective to model this. When representing certificates as GTS, we use so-called *valid histories* to determine whether a sequence matches the certificates of the other processes.

### Definition 5.1

*(Valid history)* Let $\mathcal {G}_i$ be a set of GTS. A *valid history of length* *t* *with respect to* $\mathcal {G}_i$ is a finite sequence $\sigma \in (2^V)^*$ of length *t*, where for all $g_j \in \mathcal {G}_i$, $\sigma _k \cap {O^G_{j}} = \mathrm{comp}(g_j,\hat{\sigma }\cap {I_{j}})_k \cap {O^G_{j}}$ holds for all points in time *k* with $1 \le k \le t$ and all infinite extensions $\hat{\sigma }$ of $\sigma$. The set of all valid histories of length *t* with respect to $\mathcal {G}_i$ is denoted by $\mathcal {H}^{t}_{\mathcal {G}_i}$.

Intuitively, a valid history with respect to a set $\mathcal {G}_i$ of GTS is a finite sequence that is a prefix of a computation of all GTS in $\mathcal {G}_i$. Thus, a valid history can be produced by the parallel composition of the GTS. Note that since strategies cannot look into the future, a finite word satisfies the requirements of a valid history either for all of its infinite extensions or for none of them.

**Fig. 1:**
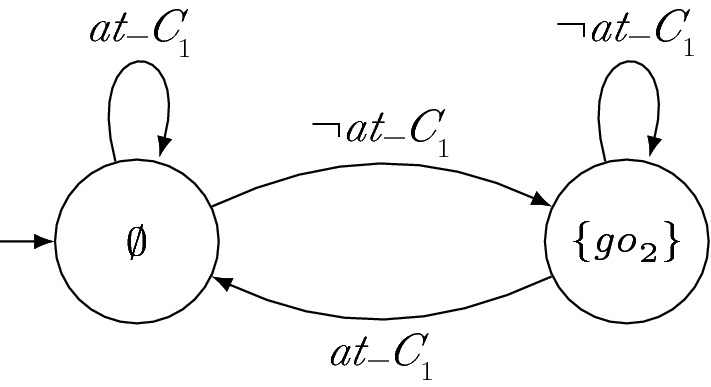
GTS for robot $r_2$. The labels of the states denote the output of the TS in the respective state

### Running example

Suppose that robot $r_2$ guarantees to give priority to $r_1$ at crossings and to move forward if $r_1$ is not at the crossing. A GTS $g_2$ for $r_2$ is depicted in Fig. 1. Since $r_2$ never outputs $\mathrm{go}_{2}$ if $r_1$ is at the crossing (left state), the finite sequence $\{ at\_c _{1} \} \{ \mathrm{go}_{2} \}$ is no valid history with respect to $\{g_2\}$. Since $r_2$ outputs $\mathrm{go}_{2}$ otherwise (right state), e.g., $\{ at\_c _{2} \} \{ \mathrm{go}_{2} \}$ is a valid history with respect to $\{g_2\}$.

Since valid histories determine whether the other processes deviate from their certificates, a strategy is required to *locally satisfy* the specification in certifying synthesis with GTS if its computation is a valid history respecting the GTS of the other processes:

### Definition 5.2

*(Local satisfaction)* Let $\mathcal {G}_i$ be a set of GTS. A strategy $s_i$ for $p_i\in P^-\!$ *locally satisfies* an LTL formula $\varphi _i$ *with respect to* $\mathcal {G}_i$, denoted $s_i \models _{\mathcal {G}_i} \varphi _i$, if $\mathrm{comp}(s_i,\gamma ) \cup \gamma ' \models \varphi _i$ holds for all $\gamma \in (2^{I_{i}})^\omega$ and $\gamma ' \in (2^{V {\setminus } {V_{i}}})^\omega$ with $\mathrm{comp}(s_i,\gamma )_{| t} \cup \gamma '_{| t} \in \mathcal {H}^{t}_{\mathcal {G}_i}$ for all *t*.

**Fig. 2:**
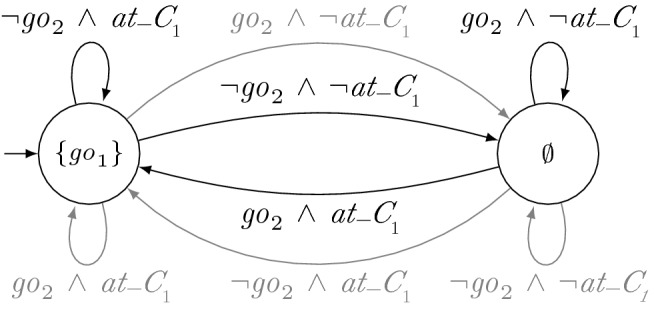
Strategy for robot $r_1$. The labels of the states denote the output of the TS in the respective state

Intuitively, requiring a strategy to locally satisfy a specification allows us to formulate the implication $\Psi _i \rightarrow \varphi _i$ used in certifying synthesis with LTL certificates also for certificates represented by GTS.

### Running example

If $r_2$ sticks to its certificate $g_2$ depicted in Fig. 1, $r_1$ can enter crossings regardless of $r_2$. Such a strategy $s_1$ for $r_1$ is shown in Fig. 2. Since neither $\sigma \,{:}= \{ at\_c _{1} \} \{ \mathrm{go}_{2} \}$ nor any finite sequence containing $\sigma$ is a valid history with respect to $g_2$, no transition for input $\mathrm{go}_{2}$ has to be considered for local satisfaction when $r_1$ is at the crossing (left state). Therefore, these transitions are depicted in gray. Similarly, no transition for $\lnot \mathrm{go}_{2}$ has to be considered when $r_1$ is not at the crossing (right state). The other transitions match valid histories and thus they are taken into account. Since no crash occurs when considering the black transitions only, $s_1 \models _{\{g_2\}} \varphi _{\mathrm{safe}} \wedge \varphi _{\mathrm{cross}_i}$ holds.

Using local satisfaction, we define certifying synthesis with GTS: Given a specification $\varphi$, certifying synthesis for $\varphi$ derives strategies $s_1, \ldots , s_n$ and *guarantee transition systems* $g_1, \ldots , g_n$ for the system processes. For all $p_i$, $s_i$ needs to locally satisfy its specification $\varphi _i$ with respect to the GTS of the other processes, i.e., $s_i \models _{\mathcal {G}_i} \varphi _i$, where $\mathcal {G}_i = \{ g_j \mid p_j\in P^-\!{\setminus }\{p_i\}\}$. To ensure that a strategy does not deviate from its certificate, $g_i$ is required to simulate $s_i$, i.e., $s_i \preceq g_i$ needs to hold.

In the following, we show that solutions of certifying synthesis with LTL certificates can be translated into solutions with GTS and vice versa. Intuitively, we construct LTL certificates from GTS that capture the exact behavior of the corresponding GTS:

### Lemma 5.1

Let $\varphi$ be an LTL formula. Let $\mathcal {S}$ and $\mathcal {G}$ be vectors of strategies and GTS for the system processes, respectively. If $(\mathcal {S},\mathcal {G})$ realizes $\varphi$, then there is a vector $\Psi$ of LTL certificates such that $(\mathcal {S},\Psi )$ realizes $\varphi$.

### Proof

Let $\mathcal {S} = \langle s_1,\ldots ,s_n \rangle$, $\mathcal {G} = \langle g_1,\ldots ,g_n \rangle$. For $p_i$, let $\mathcal {G}_i\,{:}= \{ g_j \mid p_j \in P^-\!{\setminus } \{p_i\} \}$. Suppose that $(\mathcal {S},\mathcal {G})$ realizes $\varphi$. For $p_i$, let $\psi _i$ be an LTL formula describing the exact behavior of $p_i$’s GTS $g_i$, i.e., an LTL formula with $\mathcal {L}(\psi _i) = \{ \mathrm{comp}(g_i,\gamma ) \cup \gamma ' \mid \gamma \in (2^{I_{i}})^\omega , \gamma ' \in (2^{V{\setminus }{V^G_{i}}})^\omega \}$. Since the state space of $g_i$ is finite, $\psi _{i}$ always exists. Let $\Psi \,{:}= \langle \psi _{1}, \ldots , \psi _{n} \rangle$ and $\Psi _{i}\,{:}= \{ \psi _{j} \mid p_j \in P^-\!{\setminus } \{p_i\} \}$. We claim that $(\mathcal {S},\Psi )$ realizes $\varphi$. We show that for all $p_i \in P^-\!$, $s_i \models \psi _{i} \wedge (\Psi _{i} \rightarrow \varphi _i)$ holds. Let $p_i \in P^-\!$.

First, we prove $s_i \models \psi _{i}$: Since $s_i \preceq g_i$ holds by assumption, we have $\mathrm{comp}(s_i,\gamma ) \cap {V^G_{i}} = \mathrm{comp}(g_i,\gamma )$ for all $\gamma \in (2^{I_{i}})^\omega$. Hence, by construction of $\psi _{i}$, for all $\gamma \in (2^{I_{i}})^\omega$, $\gamma ' \in (2^{V{\setminus }{V_{i}}})^\omega$, $\mathrm{comp}(s_i,\gamma ) \cup \gamma ' \in \mathcal {L}(\psi _{i})$ and thus $\mathrm{comp}(s_i,\gamma ) \cup \gamma ' \models \psi _{i}$. Therefore, $s_i \models \psi _{i}$ follows.

Next, we prove $s_i \models \Psi _{i} \rightarrow \varphi _i$. Let $\gamma \in (2^{I_{i}})^\omega$ and $\gamma ' \in (2^{V{\setminus }{V_{i}}})^\omega$. If $\mathrm{comp}(s_i,\gamma )_{| t} \cup \gamma '_{| t} \in \mathcal {H}^{t}_{\mathcal {G}_i}$ holds for all *t*, then we have $\mathrm{comp}(s_i,\gamma ) \cup \gamma ' \models \varphi _i$ since $s_i \models _{\mathcal {G}_i}\varphi _i$ holds by assumption. Thus, $\mathrm{comp}(s_i,\gamma ) \cup \gamma ' \models \Psi _{i} \rightarrow \varphi _i$. Otherwise, there is a *t* with $\mathrm{comp}(s_i,\gamma )_{| t} \cup \gamma '_{| t} \not \in \mathcal {H}^{t}_{\mathcal {G}_i}$. Let $\sigma \,{:}= \mathrm{comp}(s_i,\gamma )_{| t} \cup \gamma '_{| t}$. Then, there exists a GTS $g_j \in \mathcal {G}_i$ and an infinite extension $\hat{\sigma }$ of $\sigma$ such that we have $\sigma _k \cap {O_{j}} \ne \mathrm{comp}(g_j,\hat{\sigma }\cap {I_{j}})_k \cap {O_{j}}$ for some *k* with $1 \le k \le t$. Since strategies cannot look into the future, the above holds for *all* infinite extensions of $\sigma$ and thus in particular for $\mathrm{comp}(s_i,\gamma ) \cup \gamma '$. Hence, by construction of $\psi _{j}$, we have $\mathrm{comp}(s_i,\gamma ) \cup \gamma ' \not \in \mathcal {L}(\psi _{j})$ and therefore $\mathrm{comp}(s_i,\gamma ) \cup \gamma ' \not \in \mathcal {L}(\Psi _{i})$. Thus, $\mathrm{comp}(s_i,\gamma ) \cup \gamma ' \not \models \Psi _{i}$ and hence $\mathrm{comp}(s_i,\gamma ) \cup \gamma ' \models \Psi _{i} \rightarrow \varphi _i$ follows. Thus, $\mathrm{comp}(s'_i,\gamma )\cup \gamma ' \models \Psi _{i} \rightarrow \varphi _i$ holds for all $\gamma \in (2^{I_{i}})^\omega$, $\gamma ' \in (2^{V{\setminus }{V_{i}}})^\omega$ and therefore $s_i \models \Psi _{i} \rightarrow \varphi _i$ follows.

Hence it follows that $(\mathcal {S},\Psi )$ indeed realizes $\varphi$. $\square$

Given a solution of certifying synthesis with LTL certificates, we intuitively construct GTS that match the strategies of the given solution:

### Lemma 5.2

Let $\varphi$ be an LTL formula. Let $\mathcal {S}$ and $\Psi$ be vectors of strategies and LTL certificates, respectively, for the system processes. If $(\mathcal {S},\Psi )$ realizes $\varphi$, then there exists a vector $\mathcal {G}$ of GTS such that $(\mathcal {S},\mathcal {G})$ realizes $\varphi$.

### Proof

Let $\mathcal {S} = \langle s_1,\ldots ,s_n \rangle$, $\Psi = \langle \psi _{1},\ldots ,\psi _{n} \rangle$. For $p_i$, let $\Psi _i\,{:}= \{ \psi _{j} \mid p_j \in P^-\!{\setminus } \{p_i\}\}$. Suppose that $(\mathcal {S},\Psi )$ realizes $\varphi$. We construct a GTS $g_i$ from $s_i$: $g_i$ is a copy of $s_i$, where the labels of $g_i$ ignore outputs $v\in {O_{i}}$ that are not contained in ${O^G_{i}}$, i.e., $o^g_i(q,\varvec{i}) = o_i(q,\varvec{i}) \cap {O^G_{i}}$ for all states *q* and all inputs $\varvec{i} \in 2^{I_{i}}$, where $o^g_i$ is the labeling function of $g_i$ and $o_i$ is the labeling function of $s_i$. Let $\mathcal {G}\,{:}= \langle g_1, \ldots , g_n \rangle$ and $\mathcal {G}_i\,{:}= \{ g_j \mid p_j \in P^-\!{\setminus } \{p_i\}\}$. We claim that $(\mathcal {S}, \mathcal {G})$ realizes $\varphi$. We show that $s_i \preceq g_i$ and $s_i \models _{\mathcal {G}_i} \varphi _i$ hold for all $p_i \in P^-\!$. Let $p_i \in P^-\!$.

First, we prove $s_i \preceq g_i$: By construction of $g_i$, $s_i$ and $g_i$ only differ in their labels and the labels agree on the variables in ${O^G_{i}}$. Since the variables in ${O^G_{i}}$ are the only output variables that are shared by $s_i$ and $g_i$ and, in particular, ${O^G_{i}} \subseteq {O_{i}}$ holds, $s_i \preceq g_i$ follows.

Next, we prove $s_i \models _{\mathcal {G}_i} \varphi _i$, i.e., we show that for all $\gamma \in (2^{I_{i}})^\omega$, $\gamma ' \in (2^{V{\setminus }{V_{i}}})^\omega$ with $\mathrm{comp}(s_i,\gamma )_{| t} \cup \gamma '_{| t} \in \mathcal {H}^{t}_{\mathcal {G}_i}$ for all *t*, $\mathrm{comp}(s_i,\gamma ) \cup \gamma ' \models \varphi _i$ holds. Let $\gamma \in (2^{I_{i}})^\omega$ and $\gamma ' \in (2^{V{\setminus }{V_{i}}})^\omega$. Let $\sigma \,{:}= \mathrm{comp}(s_i,\gamma )\cup \gamma '$. By assumption, $s_i \models \psi _{i} \wedge \Psi _{i} \rightarrow \varphi _i$ and hence, in particular, $\sigma \models \Psi _{i} \rightarrow \varphi _i$ holds. If $\sigma \models \Psi _{i}$, then $\sigma \models \varphi _i$ follows. Otherwise, there is a process $p_j \in P^-\!{\setminus }\{p_i\}$ such that $\sigma \not \models \psi _{j}$. Hence, $\sigma \not \in \mathcal {L}(\psi _{j})$ holds. Then, by construction of $\psi _{j}$, we have $\sigma \ne \mathrm{comp}(g_j,\sigma \cap {I_{j}}) \cup (\sigma \cap (V {\setminus } {V^G_{j}}))$ and therefore $\sigma \cap {O^G_{j}} \ne \mathrm{comp}(g_j,\sigma \cap {I_{j}}) \cap {O^G_{j}}$ since ${O^G_{j}} \subseteq V$. Thus, there is a *k* with $\sigma _k \cap {O^G_{j}} \ne \mathrm{comp}(g_j,\sigma \cap {I_{j}})_k \cap {O^G_{j}}$ and hence $\sigma \not \in \mathcal {H}^{t}_{\{g_j\}}$ holds for all $t>k$. Since $p_j \in P^-\!{\setminus }\{p_i\}$, we have $g_j \in \mathcal {G}_j$ and thus $\mathrm{comp}(s_i,\gamma )_{| t}\cup \gamma '_{| t} \not \in \mathcal {H}^{t}_{\mathcal {G}_i}$ holds for all $t > k$. Therefore, $s_i \models _{\mathcal {G}_i} \varphi _i$ follows.

Hence, it follows that $(\mathcal {S},\mathcal {G})$ indeed realizes $\varphi$. $\square$

Since we can translate solutions of certifying synthesis with LTL certificates and solutions of certifying synthesis with GTS into each other, we can reuse the results from Sect. 4. Thus, soundness and completeness of certifying synthesis with GTS follows from Theorem 4.1 with Lemmas 5.1, 5.2:

### Theorem 5.1

Let $\varphi$ be an LTL formula. Furthermore, let $\mathcal {S} = \langle s_1, \ldots , s_n \rangle$ be a vector of strategies. Then, there exists a vector $\mathcal {G}$ of GTS such that $(\mathcal {S},\mathcal {G})$ realizes $\varphi$ if, and only if, $s_1 {{\,\mathrm{||}\,}}\cdots {{\,\mathrm{||}\,}}s_n \models \varphi$ holds.

Hence, similar to LTL certificates, certifying synthesis with GTS allows for local reasoning and thus enables modularity of the system while it still ensures that correct solutions are found for all realizable specifications. In particular, enforcing certificates to be deterministic does not rule out strategies that can be obtained with certifying synthesis with nondeterministic certificates such as certificates given by LTL formulas.

**Fig. 3:**
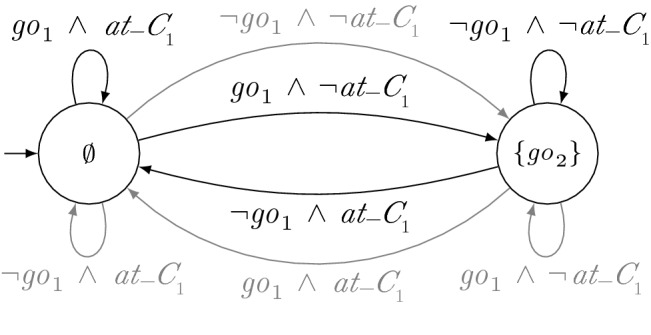
Strategy for $r_2$. The labels of the states denote the output of the TS in the respective state

**Fig. 4:**
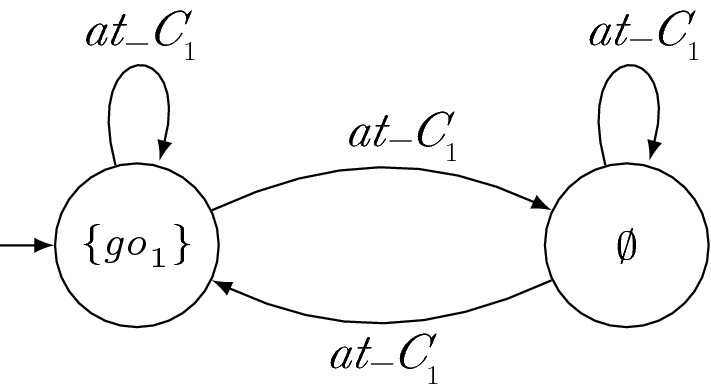
GTS for $r_1$. The labels of the states denote the output of the TS in the respective state

### Running example

**Fig. 5:**
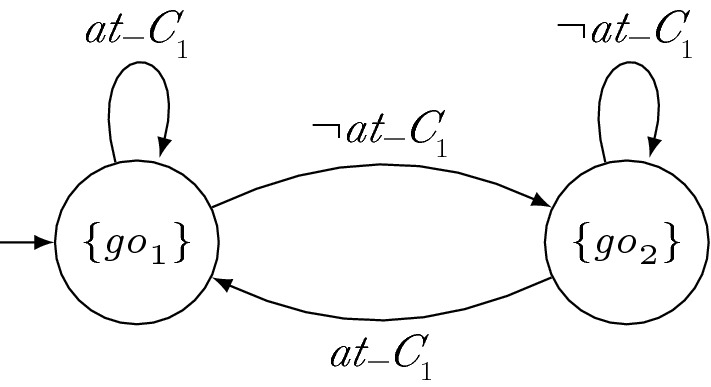
Parallel composition of the strategies $s_1$ and $s_2$ for $r_1$ and $r_2$, depicted in Figs. 2 and 3, respectively. The labels of the states denote the output of the TS in the respective state

(Synthesizing Certificates) As an example of the synthesis procedure of a distributed system with certifying synthesis and GTS, consider the manufacturing robots from Sect. 2. For simplicity, suppose that the robots do not have individual additional requirements $\varphi _{\mathrm{add}_i}$. Hence, the full specification is given by $\varphi _{\mathrm{safe}} \wedge \varphi _{\mathrm{cross}_1} \wedge \varphi _{\mathrm{cross}_2}$. Since $\mathrm{go}_{i}$ is an output variable of robot $r_i$, we obtain the subspecifications $\varphi _i = \varphi _{\mathrm{safe}} \wedge \varphi _{\mathrm{cross}_i}$. A solution of certifying synthesis is then given by the strategies depicted in Figs. 2, 3 and GTS depicted in Figs. 1 and 4. Note that $s_2$ only locally satisfies $\varphi _{\mathrm{cross}_2}$ with respect to $g_1$ when assuming that $r_1$ is not immediately again at the intersection after crossing it. However, there are solutions with slightly more complicated certificates that do not need this assumption. The parallel composition of $s_1$ and $s_2$ is depicted in Fig. 5. It is a strategy that allows $r_1$ to move forwards if it is at the crossing and that allows $r_2$ to move forwards otherwise.

## Computing relevant processes

Both variants of certifying synthesis introduced in the previous sections consider the certificates of *all* other system processes in the local objective of a process $p_i$. This is not always necessary since $\varphi _i$ might be satisfiable even if another process deviates from its guaranteed behavior. In this section, we present an optimization of certifying synthesis that reduces the number of considered certificates. For every $p_i$, we compute a set of *relevant processes* $\mathcal {R}_{i} \subseteq P^-\!{\setminus }\{p_i\}$. Certifying synthesis then only considers the certificates of the relevant processes: Let $\Psi ^\mathcal {R}_{i} = \{ \psi _{j} \in \Psi \mid p_j \in \mathcal {R}_{i} \}$, $\mathcal {G}^\mathcal {R}_i = \{ g_j \in \mathcal {G} \mid p_j \in \mathcal {R}_{i}\}$. For LTL certificates, we require $s_i \models \psi _{i} \wedge (\Psi ^\mathcal {R}_{i} \rightarrow \varphi _i)$. For GTS, $s_i \preceq g_i$ and $s_i \models _{\mathcal {G}^\mathcal {R}_i} \varphi _i$ need to hold. We denote such solutions of certifying synthesis with $(\mathcal {S},\Psi )_\mathcal {R}$ and $(\mathcal {S},\mathcal {G})_\mathcal {R}$.

The construction of the set of relevant processes $\mathcal {R}_{i}$ has to ensure that certifying synthesis is still sound and complete. In the following, we introduce a syntactic definition of relevant processes that does so. It excludes processes from $p_i$’s set of relevant processes $\mathcal {R}_{i}$ whose output variables do not occur in the subspecification $\varphi _i$:

### Definition 6.1

*(Relevant processes)* Let $\varphi$ be an LTL formula with decomposition $\langle \varphi _1,\ldots ,\varphi _n \rangle$. The *relevant processes* $\mathcal {R}_{i} \subseteq P^-\!{\setminus }\{p_i\}$ of process $p_i \in P^-\!$ are given by $\mathcal {R}_{i} = \{ p_j \in P^-\!{\setminus } \{p_i\} \mid {O_{j}} \cap {\text {prop}}(\varphi _i) \ne \emptyset \}$.

Intuitively, since ${O_{j}} \cap {\text {prop}}(\varphi _i) = \emptyset$ holds for a process $p_j \in P^-\!{\setminus } \mathcal {R}_{i}$ with $i \ne j$, $\varphi _i$ does not restrict the satisfying valuations of the output variables of $p_j$. Thus, if a sequence satisfies $\varphi _i$, then it does so for any valuations of the variables in ${O_{j}}$. Hence, the guaranteed behavior of $p_j$ does not influence the satisfiability of $\varphi _i$ and thus $p_i$ does not need to consider it:

### Theorem 6.1

Let $\varphi$ be an LTL formula. Moreover, let $\mathcal {S} = \langle s_1, \ldots , s_n \rangle$ be a vector of strategies.

1. If $(\mathcal {S},\Psi )_{\mathcal {R}}$ realizes $\varphi$ for some vector $\Psi$ of LTL certificates, then $s_1 {{\,\mathrm{||}\,}}\cdots {{\,\mathrm{||}\,}}s_n \models \varphi$. If $s_1 {{\,\mathrm{||}\,}}\cdots {{\,\mathrm{||}\,}}s_n \models \varphi$ holds, then there exist vectors $\mathcal {S}'$ and $\Psi '$ of strategies and LTL certificates such that $(\mathcal {S}',\Psi ')_{\mathcal {R}}$ realizes $\varphi$.
2. If $(\mathcal {S},\mathcal {G})_{\mathcal {R}}$ realizes $\varphi$ for some vector $\mathcal {G}$ of GTS, then $s_1 {{\,\mathrm{||}\,}}\cdots {{\,\mathrm{||}\,}}s_n \models \varphi$. If $s_1 {{\,\mathrm{||}\,}}\cdots {{\,\mathrm{||}\,}}s_n \models \varphi$ holds, then there exist vectors $\mathcal {S}'$ and $\mathcal {G}'$ of strategies and GTS such that $(\mathcal {S}',\mathcal {G}')_{\mathcal {R}}$ realizes $\varphi$.

### Proof

For both LTL certificates and GTS, soundness follows immediately from the fact that $\mathcal {R}_{i} \subseteq P^-\!{\setminus }\{p_i\}$ holds and thus we have both $\Psi ^\mathcal {R}_{i} \subseteq \Psi _{i}$ and $\mathcal {G}^{\mathcal {R}}_i \subseteq \mathcal {G}_i$.

Next, we show the completeness of certifying synthesis with LTL certificates. Suppose that $s_1 {{\,\mathrm{||}\,}}\cdots {{\,\mathrm{||}\,}}s_n \models \varphi$ holds. Then, by Theorem 4.1, there exists a vector $\Psi$ of LTL certificates such that $(\mathcal {S},\Psi )$ realizes $\varphi$. In particular, this holds for the certificates $\Psi \,{:}= \langle \psi _{1},\ldots ,\psi _{n} \rangle$ with $\mathcal {L}(\psi _i) = \{ \mathrm{comp}(s_i,\gamma )\cup \gamma ' \mid \gamma \in (2^{I_{i}})^\omega , \gamma ' \in (2^{V{\setminus }{V_{i}}})^\omega \}$. Let $\Psi _{i} = \{ \psi _{j} \mid p_j \in P^-\!{\setminus }\{p_i\} \}$. We construct strategies $s'_i$ as follows: For all $\gamma \in (2^{O_{\mathrm{env}}})^\omega$, $\gamma ' \in (2^{V{\setminus }{O_{\mathrm{env}}}})^\omega$, let $\sigma _{\gamma ,\gamma '} = (\mathrm{comp}(s_1 {{\,\mathrm{||}\,}}\cdots {{\,\mathrm{||}\,}}s_n, \gamma ) \cap {O_{i}}) \cup ((\gamma \cup \gamma ')\cap {I_{i}})$. Then, we define $\mathrm{comp}(s'_i, (\gamma \cup \gamma ') \cap {I_{i}})\,{:}= \sigma _{\gamma ,\gamma '}$. Let $\mathcal {S}'\,{:}= \langle s'_1,\ldots ,s'_n \rangle$. Let $\psi _{i}'$ be an LTL formula with $\mathcal {L}(\psi _{i}') = \{ \mathrm{comp}(s'_i,\gamma )\cup \gamma ' \mid \gamma \in (2^{I_{i}})^\omega , \gamma ' \in (2^{V{\setminus }{V_{i}}})^\omega \}$. Let $\Psi '\,{:}= \langle \psi _{1}',\ldots ,\psi _{n}' \rangle$ and $\Psi '_{i,\mathcal {R}}\,{:}= \{ \psi _{j}' \mid p_j \in \mathcal {R}_{i} \}$. Then, $(\mathcal {S}',\Psi ')_\mathcal {R}$ realizes $\varphi$. The proof is given in [15].

Next, we consider certificates represented by GTS. Suppose that $s_1 {{\,\mathrm{||}\,}}\cdots {{\,\mathrm{||}\,}}s_n \models \varphi$ holds. Let $\mathcal {S}'$ and $\Psi$ be the vectors of strategies and LTL certificates constructed as in the first part of this proof such that $(\mathcal {S}',\Psi )_\mathcal {R}$ realizes $\varphi$. Let $\Psi _{i} = \{ \psi _{j} \mid p_j \in P^-\!{\setminus } \{p_i\} \}$, $\Psi ^\mathcal {R}_{i}\,{:}= \{ \psi _{j} \mid p_j \in \mathcal {R}_{i} \}$. We construct a GTS $g_i$ as follows: $g_i$ is a copy of $s'_i$, yet, the labels of $g_i$ ignore output variables $v\in {O_{i}}$ that are not contained in ${O^G_{i}}$, i.e., $o^g_i(t,\varvec{i}) = o_i(t,\varvec{i}) \cap {O^G_{i}}$ for all states *t* and all inputs $\varvec{i} \in 2^{I_{i}}$, where $o^g_i$ is the labeling function of $g_i$ and $o_i$ is the labeling function of $s'_i$. Let $\mathcal {G}\,{:}= \langle g_1, \ldots , g_n \rangle$, $\mathcal {G}_i\,{:}= \{ g_j \mid p_j \in P^-\!{\setminus } \{p_i\}\}$, $\mathcal {G}^{\mathcal {R}}_i\,{:}= \{ g_j \mid p_j \in \mathcal {R}_{i}\}$. Then, $(\mathcal {S}',\mathcal {G})_{\mathcal {R}}$ realizes $\varphi$. For the proof, we refer to [15]. $\square$

For certifying synthesis with relevant processes, we can only guarantee that for every vector of strategies $s_1,\ldots ,s_n$ with $s_1 {{\,\mathrm{||}\,}}\cdots {{\,\mathrm{||}\,}}s_n \models \varphi$, there are *some* strategies that are part of a solution of certifying synthesis. These strategies are not necessarily $s_1, \ldots , s_n$: A strategy $s_i$ may make use of the certificate of a process $p_j$ outside of $\mathcal {R}_{i}$. That is, it may violate $\varphi _i$ on an input sequence $\gamma$ that does not stick to $g_j$ although $\varphi _i$ is satisfiable for $\gamma$. Strategy $s_i$ is not required to satisfy $\varphi _i$ on $\gamma$, a strategy that may only consider the certificates of the relevant processes, however, is. As long as the definition of relevant processes allows for finding *some* solution of certifying synthesis, like the one introduced in Definition 6.1 does as a result of Theorem 6.1, certifying synthesis is nevertheless sound and complete.

## Synthesizing certificates

In this section, we describe an algorithm for practically synthesizing strategies and certificates represented by GTS. Our approach is based on *bounded synthesis* [16] and bounds the size of the strategies and of the certificates. This allows for producing size-optimal solutions in either terms of strategies or certificates. Like for monolithic bounded synthesis [9, 16], we encode the search for a solution of certifying synthesis of a certain size into a SAT constraint system. We reuse parts of the constraint system for monolithic systems.

An essential part of bounded synthesis is to determine whether a strategy $s_i$ satisfies an LTL formula $\varphi _i$. To do so, we construct an equivalent universal co-Büchi automaton $\mathcal {A}_i$ with $\mathcal {L}(\mathcal {A}_i) = \mathcal {L}(\varphi _i)$. Then, we check whether $\mathcal {A}_i$ accepts $\mathrm{comp}(s_i,\gamma )\cup \gamma '$ for all $\gamma \in (2^{I_{i}})^\omega$, $\gamma ' \in (2^{V{\setminus }{V_{i}}})^\omega$, i.e., whether all runs of $\mathcal {A}_i$ induced by $\mathrm{comp}(s_i,\gamma )\cup \gamma '$ contain only finitely many visits to rejecting states. So far, we used local satisfaction to formalize that in compositional synthesis with GTS a strategy only needs to satisfy its specification as long as the other processes stick to their guarantees, i.e., we changed the satisfaction condition. To reuse existing algorithms for bounded synthesis, however, we now incorporate this property of certifying synthesis into the strategy instead: We utilize the observation that a finite run of an $\omega$-automaton can never visit rejecting states infinitely often. Hence, by ensuring that the automaton produces finite runs on all sequences that deviate from a certificate, checking whether a strategy satisfies a specification can still be done by checking whether the runs of the automaton induced by the computations of the strategy visit rejecting states only finitely often.

In the following, we therefore model strategies with *incomplete* transition systems. The domain of definition of their transition function is defined such that the computation of a strategy is infinite if, and only if, the other processes stick to their guarantees:

### Definition 7.1

*(Local strategy)* A *local strategy* $s_i$ for $p_i \in P^-\!$ *with respect to* a set $\mathcal {G}_i$ of GTS is represented by a TS $\mathcal {T}_i = (T,t_0,\tau ,o)$ with a partial transition function $\tau : T \times 2^{I_{i}} \rightharpoonup T$. The domain of definition of $\tau$ is defined such that $\mathrm{comp}(s_i,\gamma )$ is infinite for $\gamma \in (2^{I_{i}})^\omega$ if, and only if, there exists $\gamma ' \in (2^{V {\setminus } {V_{i}}})^\omega$ such that $\mathrm{comp}(s_i,\gamma )_{| t} \cup \gamma '_{| t} \in \mathcal {H}^{t}_{\mathcal {G}_i}$ holds for all points in time *t*.

Intuitively, a local strategy thus omits all transitions that are invoked by an input that may only occur if the other processes deviate from their certificates.

### Running example

Consider strategy $s_1$ for robot $r_1$ and GTS $g_2$ for $r_2$, depicted in Figs. 1 and 2, respectively. From $s_1$, we can construct a local strategy $s'_1$ for robot $r_1$ with respect to $\{g_2\}$ by eliminating the gray transitions.

Given a specification $\varphi$, certifying synthesis *with local strategies* derives GTS $g_1,\ldots ,g_n$ and local strategies $s_1,\ldots ,s_n$ respecting these guarantees, such that for all $p_i \in P^-\!$, $s_i \preceq g_i$ holds and all runs of $\mathcal {A}_i$ induced by $\mathrm{comp}(s_i,\gamma )\cup \gamma '$ contain only finitely many visits to rejecting states for all $\gamma \in (2^{I_{i}})^\omega$, $\gamma ' \in (2^{V {\setminus } {V_{i}}})^\omega$, where $\mathcal {A}_i$ is a universal co-Büchi automaton with $\mathcal {L}(\mathcal {A}_i)=\mathcal {L}(\varphi _i)$.

Every solution of certifying synthesis for a specification $\varphi$ with local strategies can be translated into a solution of certifying synthesis for $\varphi$ with local satisfaction and vice versa: We can *extend* the local strategy of $p_i$ with $p_i$’s guaranteed behavior, i.e., we obtain a complete strategy from the local one by reconstructing the missing transitions from the corresponding GTS. Vice versa, we can *restrict* a complete strategy obtained by certifying synthesis with local satisfaction to those transitions that match the GTS of the other processes, i.e., we delete all transitions of the strategy that can only be taken if the other processes deviate from their specifications. For the formal constructions of the translations and their proofs of correctness, we refer to [15].

Yet, local strategies obtained by restricting complete strategies only realize $\varphi$ if the satisfaction of each subspecification $\varphi _i$ solely depends on the variables that the corresponding process can observe. This is due to a slight difference in the satisfaction of $\varphi _i$ with local strategies and local satisfaction with complete strategies: The latter requires a strategy $s_i$ to satisfy $\varphi _i$ if *all processes* stick to their guarantees. The former, in contrast, requires $s_i$ to satisfy $\varphi _i$ if *all processes producing observable outputs* stick to their guarantees. Hence, if $p_i$ cannot observe whether $p_j$ deviates from its guarantee, satisfaction with local strategies requires $s_i$ to satisfy $\varphi _i$ even if $p_j$ deviates, while local satisfaction does not.

Thus, while soundness of certifying synthesis with local strategies follows from the corresponding result for certifying synthesis with local satisfaction and the fact that the parallel composition of local strategies coincides with the parallel composition of their extensions, we only obtain conditional completeness:

### Theorem 7.1

Let $\varphi$ be an LTL formula. If there are vectors $\mathcal {S} = \langle s_1,\ldots ,s_n \rangle$ and $\mathcal {G}$ of local strategies and GTS such that $(\mathcal {S},\mathcal {G})$ realizes $\varphi$, then $s_1 {{\,\mathrm{||}\,}}\cdots {{\,\mathrm{||}\,}}s_n \models \varphi$. If there is a vector $\mathcal {S} = \langle s_1,\ldots ,s_n \rangle$ of complete strategies such that $s_1 {{\,\mathrm{||}\,}}\cdots {{\,\mathrm{||}\,}}s_n \models \varphi$ and if ${\text {prop}}(\varphi _i) \subseteq {V_{i}}$ holds for all $p_i\in P^-\!$, then there are vectors $\mathcal {S}'$, $\mathcal {G}$ of local strategies and GTS such that $(\mathcal {S}',\mathcal {G})$ realizes $\varphi$.

The slight difference between local strategies and local satisfaction yielding only conditional completeness for certifying synthesis with local strategies is needed in order to technically incorporate the requirements of certifying synthesis into the strategy and thus to be able to reuse existing bounded synthesis frameworks. Although this is at general completenesses expanse, we experienced that in practice many distributed systems indeed satisfy the condition that is needed for completeness. In fact, all benchmarks described in Sect. 9 satisfy it.

**Fig. 6:**
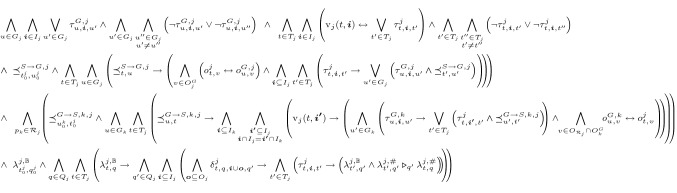
SAT constraint system $\mathcal {C}_{A,\varphi ,\mathcal {B}}$ encoding the search for a local strategy TS $\mathcal {T}_j = (T_j,t^j_0,\tau _j,o_j)$ and a guarantee TS $\mathcal {T}^G_{j} = (G_j,u^j_0,\tau ^G_j,o^G_j)$ of process $p_j$ satisfying the requirements of certifying synthesis with local strategies for $\varphi$. Variable $\delta ^{j}_{q,\varvec{i},q'}$ encodes the transition function of the universal co-Büchi automaton $\mathcal {A}_j$ for $\varphi _j$. $\tau ^{j}_{t,\varvec{i},t'}$ and ${\tau }^{G,j}_{u,\varvec{i},u'}$ encode the transition functions of the strategy and the GTS of $p_j$, respectively. $o^{j}_{t,v}$ and ${o}^{G,j}_{u,v}$ encode their labeling functions. $\lambda ^{j,\mathbb {B}}_{t,q}$ and $\lambda ^{j,\#}_{t,q}$ encode the reachability and the annotation of a state in the run graph needed for encoding the existence of a valid annotation. ${\preceq }^{S \rightarrow G,j}_{t,u}$ and ${\preceq }^{G \rightarrow S,k,j}_{u,t}$ encode the simulation relation from $\mathcal {T}_j$ to $\mathcal {T}^G_{j}$ and from $\mathcal {T}^G_{k}$ to $\mathcal {T}_j$, respectively. ${\text {v}}_{j}(t,\varvec{i})$ is syntactic sugar for $\bigwedge _{v \in {O_{\mathcal {R}_{j}}}} (v\in \varvec{i}' \leftrightarrow o^{j}_{t,v})$

We encode the search for local strategies and GTS satisfying the requirements of certifying synthesis with local strategies into a SAT constraint system:

### Theorem 7.2

Let *A* be an architecture, let $\varphi$ be an LTL formula, and let $\mathcal {B}$ be the size bounds. There is a SAT constraint system $\mathcal {C}_{A,\varphi ,\mathcal {B}}$ such that (1) if $\mathcal {C}_{A,\varphi ,\mathcal {B}}$ is satisfiable, then $\varphi$ is realizable in *A*, (2) if $\varphi$ is realizable in *A* for the bounds $\mathcal {B}$ and additionally ${\text {prop}}(\varphi _i) \subseteq {V_{i}}$ holds for all $p_i\in P^-\!$, then $\mathcal {C}_{A,\varphi ,\mathcal {B}}$ is satisfiable.

The constraint system $\mathcal {C}_{A,\varphi ,\mathcal {B}}$ consists of *n* slightly adapted copies of the SAT constraint system for monolithic systems [9, 16], one for each system process. For each copy, we add variables encoding the GTS representing the certificates as well as constraints that ensure that the local strategies and certificates indeed fulfill the conditions of certifying synthesis:

To ensure that $s_j \preceq g_j$ holds, we add a constraint that explicitly encodes the existence of a simulation relation. To encode that the local strategies respect the GTS, we need to encode that $s_j$ only produces infinite sequences if the other processes do not deviate from their guarantees. To recognize whether the other processes stick to their guarantees, we extend $s_j$ with *associated outputs*: Variables that are inputs of $p_j$ and outputs of a relevant process $p_k \in \mathcal {R}_{j}$ of $p_j$. Then, we require that $s_j$ simulates $g_k$ for every relevant process $p_k \in \mathcal {R}_{j}$ of $p_j$ with respect to the variables in ${I_{j}} \cap {O_{k}}$. Note that we use a slightly more general definition of simulation here than introduced in the preliminaries: We do not require that ${I_{j}} = {I_{k}}$ and ${O_{j}} \subseteq {O_{k}}$ holds. Instead, we only consider successors for inputs that agree on shared variables and that may occur if the other processes stick to their guarantees. Then, we only require that the successors agree on the variables in ${I_{j}} \cap {O_{k}}$. Moreover, we add a constraint encoding that the transition system representing $s_i$ only has an outgoing transition with a certain input if the valuations of $s_i$’s associated outputs in it match the valuations of $s_i$’s associated outputs in the current state. The constraint system $\mathcal {C}_{A,\varphi ,\mathcal {B}}$ is presented in Fig. 6. A more detailed description of the single constraints is given in [15].

**Fig. 7:**
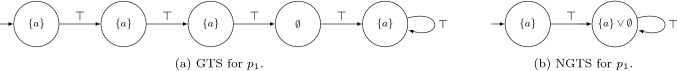
GTS and NGTS for $p_1$. The labels of the states denote the output of the TS in the respective state

Note that we build a *single* constraint system for the whole certifying synthesis task. Thus, the strategies and certificates of the individual processes are not synthesized completely independently. This is one of the main differences of our approach to the negotiation-based assume-guarantee synthesis algorithm [21]. While this prevents separate synthesis tasks and thus parallelizability, it eliminates the need for a negotiation between the processes. Moreover, it allows for completeness of the synthesis algorithm. Although the synthesis tasks are not fully separated, the constraint system $\mathcal {C}_{A,\varphi ,\mathcal {B}}$ is in most cases still significantly smaller and easier to solve than the one of classical distributed synthesis.

## Adding nondeterminism to GTS

We focused on certifying synthesis with guarantee transition systems in the previous two sections, and in [14], since representing certificates with GTS ensures deterministic guarantees. This avoids the blowup of determinizing the certificate in order to check whether a strategy satisfies its own certificate. In this section, we investigate the effect of permitting nondeterminism in GTS. We first define nondeterministic guarantee transition systems (NGTS), adapt the notion of valid histories to NGTS, and define certifying synthesis with NGTS, both in the setting with local satisfaction and with local strategies. We establish soundness and completeness of certifying synthesis with NGTS. Moreover, we present the changes in the constraint system $\mathcal {C}_{A,\varphi ,\mathcal {B}}$ that are needed to search for NGTS instead of GTS. Lastly, we discuss the advantages and disadvantages of permitting nondeterminism in the GTS.

We model the certificate $g_i$ of a process $p_i$ as a *nondeterministic* TS $\mathcal {T}^G_{n,i} = (T, t_0, \tau , o)$, called *nondeterministic guarantee transition system* (NGTS), over inputs $I_i$ and guarantee outputs $O^G_i \subseteq O_i$. The NGTS is nondeterministic in the sense that both nondeterministic transitions and nondeterministic labelings are allowed. Thus, $\tau : T \times 2^I_i \times T$ is a transition *relation* and $o: T \times 2^{O^G_i}$ is a labeling *relation*. A nondeterministic TS $\mathcal {T}_1 = (T_1,t^1_0,\tau _1,o_1)$ over *I* and $O_1$ *simulates* a deterministic TS $\mathcal {T}_2 = (T_2,t^2_0,\tau _2,o_2)$ over *I* and $O_2$ with $O_1 \subseteq O_2$, denoted $\mathcal {T}_2 \preceq \mathcal {T}_1$, if there is a simulation relation $R: T_2 \times T_1$ with $(t^2_0,t^1_0)\in R$, $\forall (t_2,t_1) \in R.~ (t_1,o_2(t_2) \cap O_1) \in o_1$, $\forall t'_2 \in T_2. \forall \varvec{i} \in 2^I.$ $(t_2,\varvec{i},t'_2) \in \tau _2 \rightarrow (\exists t'_1 \in T_1.~ \tau _1(t_1,\varvec{i}) = t'_1 \wedge (t'_2,t'_1) \in R)$. The *computation* $\mathrm{comp}_{n}(g_j,\gamma )$ *of a NGTS* $g_i$ *on input* $\gamma \in (2^{I_{i}})^\omega$ is the *set of traces* produced by $g_j$ on $\gamma$.

For NGTS, valid histories are similar to those for GTS. Yet, we require that *one* trace in the computation of the NGTS satisfies the requirements:

### Definition 8.1

*(Valid history for NGTS)* Let $\mathcal {G}_i$ be a set of NGTS. A *valid history of length* *t* *with respect to* $\mathcal {G}_i$ is a finite sequence $\sigma \in (2^V)^*$ of length *t*, where for all $g_j \in \mathcal {G}_i$, there is some $\sigma ' \in \mathrm{comp}_{n}(g_j,\hat{\sigma }\cap {I_{j}})$ such that $\sigma _k \cap {O^G_{j}} = \sigma '_k \cap {O^G_{j}}$ holds for all *k* with $1 \le k \le t$ and all infinite extensions $\hat{\sigma }$ of $\sigma$. $\mathcal {H}^{t}_{\mathcal {G}_i}\!$ denotes the set of all valid histories of length *t* with respect to $\mathcal {G}_i$.

Utilizing valid histories for NGTS, the definition of certifying synthesis with GTS directly carries over to NGTS: For $\varphi$, we derive strategies $s_1, \ldots , s_n$ and NGTS $g_1, \ldots , g_n$ such that both $s_i \models _{\mathcal {G}_i} \varphi _i$ and $s_i \preceq g_i$ hold for all $p_i \in P^-\!$. Soundness and completeness for NGTS then follows from Theorem 5.1 and the fact that we can resolve the nondeterministic choices in an NGTS while maintaining the properties of certifying synthesis:

### Theorem 8.1

Let $\varphi$ be an LTL formula. Furthermore, let $\mathcal {S} = \langle s_1, \ldots , s_n \rangle$ be a vector of strategies for the system processes. Then, there exists a vector $\mathcal {G}$ of NGTS such that $(\mathcal {S},\mathcal {G})$ is a solution of certifying synthesis for $\varphi$ if, and only if, $s_1 {{\,\mathrm{||}\,}}\cdots {{\,\mathrm{||}\,}}s_n \models \varphi$ holds.

### Proof

Since every GTS is an NGTS as well, completeness follows immediately from Theorem 5.1. Next, suppose that there is a vector $\mathcal {G} = \langle g_1,\ldots ,g_n \rangle$ of NGTS such that $(\mathcal {S},\mathcal {G})$ realizes $\varphi$. Let $g'_i$ be the GTS obtained by resolving nondeterminism in $g_i$ while maintaining the simulation requirements: $g'_i$ is a copy of $g_i$, yet, in every state we keep only a single label and, for every input, a single outgoing edge that satisfies the simulation requirements. Since $s_i \preceq g_i$ holds by assumption, such a GTS always exists. Let $\mathcal {G}'\,{:}= \langle g'_1,\ldots ,g'_n \rangle$ and $p_i \in P^-\!$. By construction of $g'_i$, $s_i \preceq g'_i$ holds and $s_i \models _{\mathcal {G}'_i} \varphi _i$ since all valid histories with respect to $\mathcal {G}'_i$ are valid histories with respect to $\mathcal {G}_i$ as well. Thus, $(\mathcal {S},\mathcal {G}')$ realizes $\varphi$ and hence $s_1 {{\,\mathrm{||}\,}}\cdots {{\,\mathrm{||}\,}}s_n \models \varphi$ follows with Theorem 5.1. $\square$

Nondeterministic GTS are particularly beneficial if a process only needs information about the behavior of another process in a certain step. For instance, consider a system with two processes $p_1$, $p_2$ with specifications  and $\varphi _2 = a \leftrightarrow b$, where ${O_{1}} = {I_{2}} = \{a\}$, ${I_{1}} = {O_{2}} = \{b\}$. Process $p_2$ only needs information about $p_1$’s behavior in the very first step, the other steps are irrelevant. The certificates for $p_1$ with GTS and NGTS are depicted in Fig. 7. With deterministic GTS, $p_1$’s certificate consists of five states, while the NGTS only has two states. Thus, the certificate size can be reduced significantly for certain specifications when using NGTS.

Moreover, permitting nondeterminism compensates overapproximations in the set of relevant processes: If knowledge on the behavior of a process is not needed although it is relevant according to the syntactic criterion stated in Definition 6.1, we can derive a single-state NGTS, while a deterministic GTS needs to describe the processes’ full behavior on the guarantee outputs.

Similar to Theorem 8.1, certifying synthesis with GTS and local strategies carries over to NGTS. With the very same construction as for local satisfaction, soundness and conditional completeness follows:

### Theorem 8.2

Let $\varphi$ be an LTL formula. If there are vectors $\mathcal {S} = \langle s_1,\ldots ,s_n \rangle$ and $\mathcal {G}$ of local strategies and NGTS such that $(\mathcal {S},\mathcal {G})$ realizes $\varphi$, then ${s_1 {{\,\mathrm{||}\,}}\cdots {{\,\mathrm{||}\,}}s_n \!\models \varphi }$. If there is a vector $\mathcal {S} = \langle s_1,\ldots ,s_n \rangle$ of complete strategies such that $s_1 {{\,\mathrm{||}\,}}\cdots {{\,\mathrm{||}\,}}s_n \models \varphi$ and if ${\text {prop}}(\varphi _i) \subseteq {V_{i}}$ holds for all $p_i\in P^-\!$, then there are vectors $\mathcal {S}'$, $\mathcal {G}$ of local strategies and NGTS such that $(\mathcal {S}',\mathcal {G})$ realizes $\varphi$.

To practically synthesize a solution of certifying synthesis with NGTS, we encode the search for strategies and certificates into a SAT constraint system. We reuse most of the constraint system for certifying synthesis with GTS shown in Fig. 6, and only need to adapt a few variable encodings and constraints: The labeling function of the NGTS in state *u* for output *v* is not encoded by a single variable ${o}^{G,j}_{u,v}$ anymore, but with two variables denoting that *v* is $\mathrm{true}$ or $\mathrm{false}$ in *u*, respectively, enabling that *v* can be both $\mathrm{true}$ and $\mathrm{false}$. All constraints using the labeling function are adapted accordingly. Moreover, we drop the constraint encoding that there is at most one outgoing edge for every state and input, i.e., the constraint encoding determinism.

Due to the nondeterminism in the GTS, the search space clearly gets larger. However, it also allows for certificates with fewer states, enabling us to find solutions with smaller bounds and thus to reduce the search space again. To investigate this trade-off on different benchmarks, we compare certifying synthesis with GTS and NGTS experimentally in the next section.

## Experimental results

We have implemented certifying synthesis with local strategies and both deterministic and nondeterministic GTS. Our implementation expects an LTL formula and its decomposition as well as the system architecture and bounds on the strategy and certificate sizes as input. Specification decomposition can easily be automated by, e.g., implementing Definition 4.1. Our implementation extends the bounded synthesis tool BoSy [10] for monolithic systems to certifying synthesis for distributed systems. In particular, we extend and adapt BoSy’s SAT encoding [9] as described in Sect. 7.

**Table 1:** Results on scalable benchmarks

Benchmark	Par.	Cert.	Dist.	Dom.
n-ary Latch	2	**0**.**89**	41.26	4.75
	3	**0**.**91**	TO	6.40
	4	**0**.**92**	TO	8.46
	5	**0**.**94**	TO	10.74
	6	**12**.**26**	TO	13.89
	7	105.69	TO	**15**.**06**
Gen. Buff.	1	**1**.**20**	6.59	5.23
	2	**2**.**72**	3012.51	10.53
	3	**122**.**09**	TO	961.60
Load Bal.	1	**0**.**98**	1.89	2.18
	2	**1**.**64**	2.39	–
Shift	2	**1**.**10**	1.99	4.76
	3	**1**.**13**	4.16	7.04
	4	**1**.**14**	TO	11.13
	5	**1**.**29**	TO	13.68
	6	**2**.**20**	TO	16.01
	7	**9**.**01**	TO	16.08
	8	71.89	TO	**19**.**38**
R.-C. Adder	1	**0**.**878**	1.83	–
	2	**2**.**09**	36.84	–
	3	**106**.**45**	TO	–

Reported is the parameter (Par.) and the running time in seconds for certifying synthesis (Cert.), distributed BoSy (Dist.), and dominant strategy synthesis (Dom.)

Bold values highlight the lowest (and thus best) running time in the comparison depicted

**Table 2:** Results for the *manufacturing robots* benchmark

	Strategy size			
Par.	Cert.	Dist.	Cert.	Dist.
2, 3	2, 6	6	**1**.**59**	2.91
2, 4	2, 4	4	**1**.**18**	2.43
2, 5	2, 10	10	**3**.**97**	299.11
2, 6	2, 6	6	**1**.**40**	3.25
2, 7	2, 14	14	**76**.**32**	TO
2, 8	2, 8	8	**2**.**47**	5.28
2, 9	2, 18	18	**1832**.**53**	TO
2, 10	2, 10	10	**7**.**78**	106.34
3, 4	6, 4	12	**1**.**44**	TO
3, 5	6, 10	30	**32**.**83**	TO
3, 6	6, 6	6	**2**.**04**	3.43
3, 7	6, 14	42	**373**.**90**	TO
3, 8	6, 8	24	**8**.**82**	TO
3, 9	6, 18	18	TO	TO
3, 10	6, 10	30	**30**.**92**	TO
4, 5	4, 10	20	**11**.**66**	TO
4, 6	4, 6	12	**2**.**04**	TO
4, 7	4, 14	28	**221**.**17**	TO
4, 8	4, 8	8	**3**.**28**	6.06
4, 9	4, 18	36	**2911**.**26**	TO
4, 10	4, 10	20	**7,93**	TO
5, 6	10, 6	30	**26**.**16**	TO
5, 7	10, 14	35	TO	TO
5, 8	10, 8	40	**26**.**164**	TO
5, 9	10, 18	45	TO	TO
5, 10	10, 10	10	**89**.**87**	335.98

Reported are the parameters (Par.), the implementation sizes of certifying synthesis (Cert.) and distributed BoSy (Dist.), and the running time in seconds

Bold values highlight the lowest (and thus best) running time in the comparison depicted

We compare our deterministic approach to two extensions of BoSy: One for distributed systems [2] and one for synthesizing dominant process strategies separately, implementing the compositional synthesis algorithm presented in [7]. The results are shown in Tables 1 and 2. We used a machine with a 3.1 GHz Dual-Core Intel Core i5 processor and 16 GB of RAM, and a timeout of 60 min. We use the SMT encoding of distributed BoSy since the other ones either do not support most of our architectures (QBF), or cause memory errors frequently (SAT). Since the running times of the underlying SMT solver vary immensely, we report on the average running time of 10 runs. Synthesizing dominant strategies separately is incomplete and thus we cannot report on results for all benchmarks. We could not compare our algorithm to the iterative distributed synthesis tool Agnes [21], since it currently does not support most of our architectures or specifications.

Four benchmarks stem from the synthesis competition [17]. The latch is parameterized in the number of bits, the generalized buffer in the number of senders, the load balancer in the number of servers, and the shift in the number of inputs. The fifth benchmark, a ripple-carry adder, is parameterized in the number of bits. The last benchmark describes the robots from Sect. 2 and is parameterized in the size of the objectives $\varphi _{\mathrm{add}_i}$. The system architectures are given in [15].

For the latch, the generalized buffer, the ripple-carry adder, and the shift, certifying synthesis clearly outperforms distributed BoSy. For many parameters, the latter does not terminate within 60 min, while certifying synthesis solves the tasks in less than 13 s. Here, a process does not need to know the full behavior of the relevant processes. Thus, the certificates are notably smaller than the strategies. A process of the adder, for instance, only needs information about the carry bit of the previous process, the sum bit is irrelevant.

In contrast, the load balancer requires the certificates to contain the full behavior of the processes. Thus, the benefit of the compositional approach lies solely in the specification decomposition. This advantage suffices to produce a solution faster than distributed BoSy. Yet, for other benchmarks with full certificates, the overhead of synthesizing certificates dominates the benefit of specification decomposition for larger parameters, showcasing that certifying synthesis is particularly beneficial if a small interface between the processes exists.

The robot benchmark is designed such that the interface stays small for all parameters. Thus, it demonstrates the advantage of abstracting away irrelevant behavior. We scale $\varphi _{\mathrm{add}_i}$, while $\varphi _{\mathrm{safe}}$ and $\varphi _{\mathrm{cross}_i}$ are not changed: $k_i$ denotes that $r_i$ needs to visit a machine in every $k_i$th step. Certifying synthesis clearly outperforms distributed BoSy on all instances. The size of the solutions of certifying synthesis only depends on the parameter of the respective robot and the size of the other robot’s certificate, which is two for all parameters, while the size of the solution with distributed BoSy depends on the parameters for *both* robots. Therefore, the solution sizes and thus the running times do not grow in parallel. This demonstrate that certifying synthesis is extremely beneficial for specifications where small certificates exist. This directly corresponds to the existence of a small interface between the processes of the system. Hence, bounding the size of the certificates indeed guides the synthesis procedure in finding solutions fast.

The weaker winning condition *dominance* [6] poses implicit assumptions on the behavior of the other processes. These assumptions do not always suffice: There are no independent dominant strategies for the load balancer, the ripple-carry adder, and the robots. While certifying synthesis performs better for the generalized buffer, the overhead of synthesizing explicit certificates becomes clear for the latch and the shift: For larger parameters, synthesizing dominant strategies outperforms certifying synthesis. However, the implicit assumptions do not encapsulate the required interface between the processes and thus they do not increase the understandability of the system’s interconnections.

**Table 3:** Comparison of GTS and NGTS

	Strat. and Cert. Size			
Par.	GTS	NGTS	GTS	NGTS
3	6, 6-3	4, 3-2	1.30	**1**.**03**
4	4, 4-4	4, 2-2	1.17	**0**.**96**
5	10, 10-5	6, 3-2	31.67	**1**.**25**
6	6, 6-6	6, 2-2	4.35	**1**.**01**
7	14, 14-7	8, 3-2	TO	**1**.**23**
8	8, 8-8	8, 2-2	TO	**1**.**34**
9	18, 18-9	10, 3-2	TO	**1**.**91**
10	10, 10-10	10, 2-2	TO	**1**.**30**
11	22, 22-11	12, 3-2	TO	**3**.**44**
12	12, 12-12	12, 2-2	TO	**3**.**34**
13	26, 26-13	14, 3-2	TO	**13**.**88**
14	14, 14-14	14, 2-2	TO	**10**.**52**
15	30, 30-15	16, 3-2	TO	**30**.**48**
16	16, 16-16	16, 2-2	TO	**28**.**86**
17	34, 34-17	18, 3-2	TO	**398**.**56**
18	18, 18-18	18, 2-2	TO	**168**.**19**
19	38, 38-19	20, 3-2	TO	**299**.**80**
20	20, 20-20	20, 2-2	TO	**428**.**82**

Reported is the parameter, the strategy and certificate sizes (of the form: size strat. $p_1$, size strat. $p_2$ – size cert. $p_1$), and the running time in seconds. For the second process, the certificate is of size 2 for all parameters

Bold values highlight the lowest (and thus best) running time in the comparison depicted

In a third line of experiments, we compared our implementations with GTS and NGTS. We synthesized solutions with NGTS for the benchmarks presented in Table 1. Note that there is no smaller NGTS for any of these benchmarks, i.e., there is no advantage in permitting nondeterminism. In contrast to GTS, the running times vary widely with NGTS. Most likely, permitting nondeterminism increases the degree of freedom and thus the possibility for the underlying SAT solver to “take a wrong path”, yielding the varying running times. Hence, we consider the average running time over 10 runs. For smaller parameters, the running times of certifying synthesis with NGTS are similar to those with GTS. For larger parameters, the overhead increases: For the 7-ary latch, we have an overhead of 12%, for the generalized parameter with three senders of 11%, and for the shift with eight inputs of 23%.

To analyze the advantage of nondeterministic certificates, we consider a benchmark with two processes, where, similar to the example in Sect. 8, the NGTS for $p_1$ stays small, while the size of the GTS increases with the parameter. Here, due to the larger GTS certificate the strategy sizes for both $p_1$ and $p_2$ also increase. The results are shown in Table 3. Permitting nondeterminism has a clear benefit on the running time: With GTS, certifying synthesis does not terminate within 60 min from $k=7$ on, while we still synthesize a solution with NGTS in less than 8 min up to $k=20$. The fact that not only the certificate sizes but also the strategy sizes increase for GTS has a great impact on this significant difference. For benchmarks where only the certificate sizes differ, the running times do not differ as much. Oftentimes, however, large certificates yield an increase in the strategy size as well. Hence, the experiment demonstrates again that certifying synthesis strives when solutions with small certificates exist.

## Conclusion

We have presented a sound and complete synthesis algorithm that reduces the complexity of distributed synthesis by decomposing the global specification into local requirements on the individual processes. It synthesizes additional certificates that capture a certain behavior a process commits to. The certificates then form an assume-guarantee contract, allowing a process to rely on the other processes to not deviate from their guaranteed behavior. The certificates increase the understandability of the system and the solution since the certificates capture which agreements the processes have to establish. Moreover, the certificates form a contract between the processes: The synthesized strategies can be exchanged safely as long as the new strategy still complies with the contract, i.e., as long as it does not deviate from the certificate, enabling modularity.

We have introduced two representations of the certificates, as LTL formulas and as labeled transition systems. For the latter one, we presented an encoding of the search for strategies and certificates into a SAT constraint solving problem. Moreover, we have introduced a technique for reducing the number of certificates that a process needs to consider by determining relevant processes. We have implemented the certifying synthesis algorithm and compared it to two extensions of the synthesis tool BoSy to distributed systems. Furthermore, we analyzed the advantage of permitting nondeterminism in the certificates. The results clearly show the advantage of compositional approaches as well as of guiding the synthesis procedure by bounding the size of the certificates: For benchmarks where small interfaces between the processes exist, certifying synthesis outperforms the other algorithms significantly. If no solution with small interfaces exist, the overhead of certifying synthesis is small. Permitting nondeterminism can reduce the strategy and certificate sizes significantly.

## References

[1] Alur R, Moarref S, Topcu U (2015) Pattern-based refinement of assume-guarantee specifications in reactive synthesis. In: Baier C, Tinelli C (eds) Tools and algorithms for the construction and analysis of systems - 21st international conference, TACAS 2015, held as part of the European joint conferences on theory and practice of software, ETAPS 2015, London, UK, April 11-18, 2015. Proceedings, Lecture Notes in Computer Science, vol. 9035, pp. 501–516. Springer. 10.1007/978-3-662-46681-0_49

[2] Baumeister JE (2017) Encodings of bounded synthesis for distributed systems. Bachelor’s thesis, Saarland University

[3] Bloem R, Chatterjee K, Jacobs S, Könighofer R (2015) Assume-guarantee synthesis for concurrent reactive programs with partial information. In Tools and algorithms for the construction and analysis of systems - 21st international conference, TACAS 2015, held as part of the European joint conferences on theory and practice of software, ETAPS 2015, London, UK, April 11-18, 2015. Proceedings, Lecture Notes in Computer Science, vol. 9035, pp 517–532. Springer. 10.1007/978-3-662-46681-0_50

[4] Brenguier R, Raskin J, Sankur O (2017). Assume-admissible synthesis. Acta Inform.

[5] Chatterjee K, Henzinger TA (2007) Assume-guarantee synthesis. In: Grumberg O, Huth M (eds) Tools and algorithms for the construction and analysis of systems, 13th international conference, TACAS 2007, held as part of the joint European conferences on theory and practice of software, ETAPS 2007 Braga, Portugal, March 24 - April 1, 2007, Proceedings, Lecture Notes in Computer Science, vol. 4424, pp 261–275. Springer. 10.1007/978-3-540-71209-1_21

[6] Damm W, Finkbeiner B (2011) Does it pay to extend the perimeter of a world model? In FM 2011: formal methods - 17th international symposium on formal methods, Limerick, Ireland, June 20-24, 2011. Proceedings, Lecture Notes in Computer Science, vol. 6664, pp 12–26. Springer. 10.1007/978-3-642-21437-0_4

[7] Damm W, Finkbeiner B (2014) Automatic compositional synthesis of distributed systems. In: Jones CB, Pihlajasaari P, Sun J (eds) FM 2014: formal methods - 19th international symposium, Singapore, May 12-16, 2014. Proceedings, Lecture Notes in Computer Science, vol. 8442, pp 179–193. Springer. 10.1007/978-3-319-06410-9_13

[8] de Roever WP, Langmaack H, Pnueli A (eds) (1998) Compositionality: the significant difference, international symposium, COMPOS’97, Bad Malente, Germany, September 8-12, 1997. Revised Lectures, Lecture Notes in Computer Science, vol. 1536. Springer. 10.1007/3-540-49213-5

[9] Faymonville P, Finkbeiner B, Rabe MN, Tentrup L (2017) Encodings of bounded synthesis. In: Legay A, Margaria T (eds) Tools and algorithms for the construction and analysis of systems - 23rd international conference, TACAS 2017, held as part of the European joint conferences on theory and practice of software, ETAPS 2017, Uppsala, Sweden, April 22-29, 2017, Proceedings, Part I, Lecture Notes in Computer Science, vol. 10205, pp 354–370. 10.1007/978-3-662-54577-5_20

[10] Faymonville P, Finkbeiner B, Tentrup L (2017) BoSy: an experimentation framework for bounded synthesis. In: Majumdar R, Kuncak V (eds) Computer aided verification - 29th international conference, CAV 2017, Heidelberg, Germany, July 24-28, 2017, Proceedings, Part II, Lecture Notes in Computer Science, vol. 10427, pp 325–332. Springer. 10.1007/978-3-319-63390-9_17

[11] Filiot E, Jin N, Raskin J (2010) Compositional algorithms for LTL synthesis. In: Bouajjani A, Chin W(eds) Automated technology for verification and analysis - 8th international symposium, ATVA 2010, Singapore, September 21-24, 2010. Proceedings, Lecture Notes in Computer Science, vol. 6252, pp 112–127. Springer. 10.1007/978-3-642-15643-4_10

[12] Finkbeiner B, Geier G, Passing N (2021) Specification decomposition for reactive synthesis. In: Dutle A, Moscato MM, Titolo L, Muñoz CA, Perez I (eds) NASA formal methods - 13th international symposium, NFM 2021, virtual event, May 24-28, 2021, Proceedings, Lecture Notes in Computer Science, vol. 12673, pp 113–130. Springer. 10.1007/978-3-030-76384-8_8

[13] Finkbeiner B, Passing N (2020) Dependency-based compositional synthesis. In: Hung DV, Sokolsky O (eds) Automated technology for verification and analysis - 18th international symposium, ATVA 2020, Hanoi, Vietnam, October 19-23, 2020, Proceedings, Lecture Notes in Computer Science, vol. 12302, pp 447–463. Springer. 10.1007/978-3-030-59152-6_25

[14] Finkbeiner B, Passing N (2021) Compositional synthesis of modular systems. In: Automated technology for verification and analysis - 19th international symposium, ATVA 2021, Gold Coast, Australia, October 18-22, 2021

[15] Finkbeiner B, Passing N (2021) Compositional synthesis of modular systems (Full Version). CoRR arXiv: abs/2106.14783 10.1007/s11334-022-00450-w PMC9468117 36118299

[16] Finkbeiner B, Schewe S (2013) Bounded synthesis. STTT pp 519–539

[17] Jacobs S, Bloem R, Colange M, Faymonville P, Finkbeiner B, Khalimov A, Klein F, Luttenberger M, Meyer PJ, Michaud T, Sakr M, Sickert S, Tentrup L, Walker A (2019) The 5th reactive synthesis competition (SYNTCOMP 2018): benchmarks, participants & results. CoRR arXiv:abs/1904.07736

[18] Kugler H, Segall I (2009) Compositional synthesis of reactive systems from live sequence chart specifications. In: Kowalewski S, Philippou A (eds) Tools and algorithms for the construction and analysis of systems, 15th international conference, TACAS 2009, held as part of the joint European conferences on theory and practice of software, ETAPS 2009, York, UK, March 22-29, 2009. Proceedings, Lecture Notes in Computer Science, vol. 5505, pp 77–91. Springer. 10.1007/978-3-642-00768-2_9

[19] Kupferman O, Piterman N, Vardi MY (2006) Safraless compositional synthesis. In: Ball T, Jones RB (eds) Computer aided verification, 18th international conference, CAV 2006, Seattle, WA, USA, August 17-20, 2006, Proceedings, Lecture Notes in Computer Science, vol. 4144, pp 31–44. Springer. 10.1007/11817963_6

[20] Kupferman O, Vardi MY (2005) Safraless decision procedures. In: 46th annual IEEE symposium on foundations of computer science (FOCS 2005), 23-25 October 2005, Pittsburgh, PA, USA, Proceedings, pp 531–542. IEEE Computer Society. 10.1109/SFCS.2005.66

[21] Majumdar R, Mallik K, Schmuck A, Zufferey D (2020). Assume-guarantee distributed synthesis. IEEE Trans Comput Aided Des Integr Circuits Syst..

[22] Pnueli A (1977) The temporal logic of programs. In: 18th annual symposium on foundations of computer science, Providence, Rhode Island, USA, 31 October - 1 November 1977, pp 46–57. IEEE Computer Society. 10.1109/SFCS.1977.32

[23] Safra S (1988) On the complexity of omega-automata. In: 29th annual symposium on foundations of computer science, White Plains, New York, USA, 24-26 October 1988, pp. 319–327. IEEE Computer Society. 10.1109/SFCS.1988.21948

